# The correlation between the Th17/Treg cell balance and bone health

**DOI:** 10.1186/s12979-020-00202-z

**Published:** 2020-10-14

**Authors:** Lei Zhu, Fei Hua, Wenge Ding, Kai Ding, Yige Zhang, Chenyang Xu

**Affiliations:** The Third Affiliated Hospital of Soochow University, The First People’s Hospital of Changzhou, Jiangsu, 213003 China

**Keywords:** Regulatory T cells, Helper T cell 17, Balance, Osteoclasts, Osteoporosis, Bone immunology

## Abstract

With the ageing of the world population, osteoporosis has become a problem affecting quality of life. According to the traditional view, the causes of osteoporosis mainly include endocrine disorders, metabolic disorders and mechanical factors. However, in recent years, the immune system and immune factors have been shown to play important roles in the occurrence and development of osteoporosis. Among these components, regulatory T (Treg) cells and T helper 17 (Th17) cells are crucial for maintaining bone homeostasis, especially osteoclast differentiation. Treg cells and Th17 cells originate from the same precursor cells, and their differentiation requires involvement of the TGF-β regulated signalling pathway. Treg cells and Th17 cells have opposite functions. Treg cells inhibit the differentiation of osteoclasts in vivo and in vitro, while Th17 cells promote the differentiation of osteoclasts. Therefore, understanding the balance between Treg cells and Th17 cells is anticipated to provide a new idea for the development of novel treatments for osteoporosis.

## Introduction

Osteoporosis is a systemic bone disease characterized by a decrease in the bone mineral content and destruction of the bone microstructure, which increases the fragility of bone and the incidence of fracture [1]. According to the traditional view, the occurrence of osteoporosis is associated with endocrine disorders, metabolic disorders and mechanical factors, especially oestrogen deficiency. However, osteoporosis is also considered a chronic inflammatory bone disease [2]. In recent years, research on the pathogenesis of osteoporosis has been extended to address the interaction between the skeletal system and the immune system. Many studies have demonstrated that immune disorders can cause many skeletal diseases [3]. Since Arron and Choi proposed the concept of osteoimmunology in 2000, this cross-disciplinary field has attracted great interest and attention [4].

In this review, we introduce the correlation between bone loss and Treg cells as well as Th17 cells. In addition, the impact of the balance between Treg cells and Th17 cells on osteoporosis is presented. Moreover, we summarize the relevant factors that affect the Th17/Treg cell balance, aiming to provide new ideas for the treatment of osteoporosis in the future.

### Immunological factors of osteoporosis

**Fig. 1:**
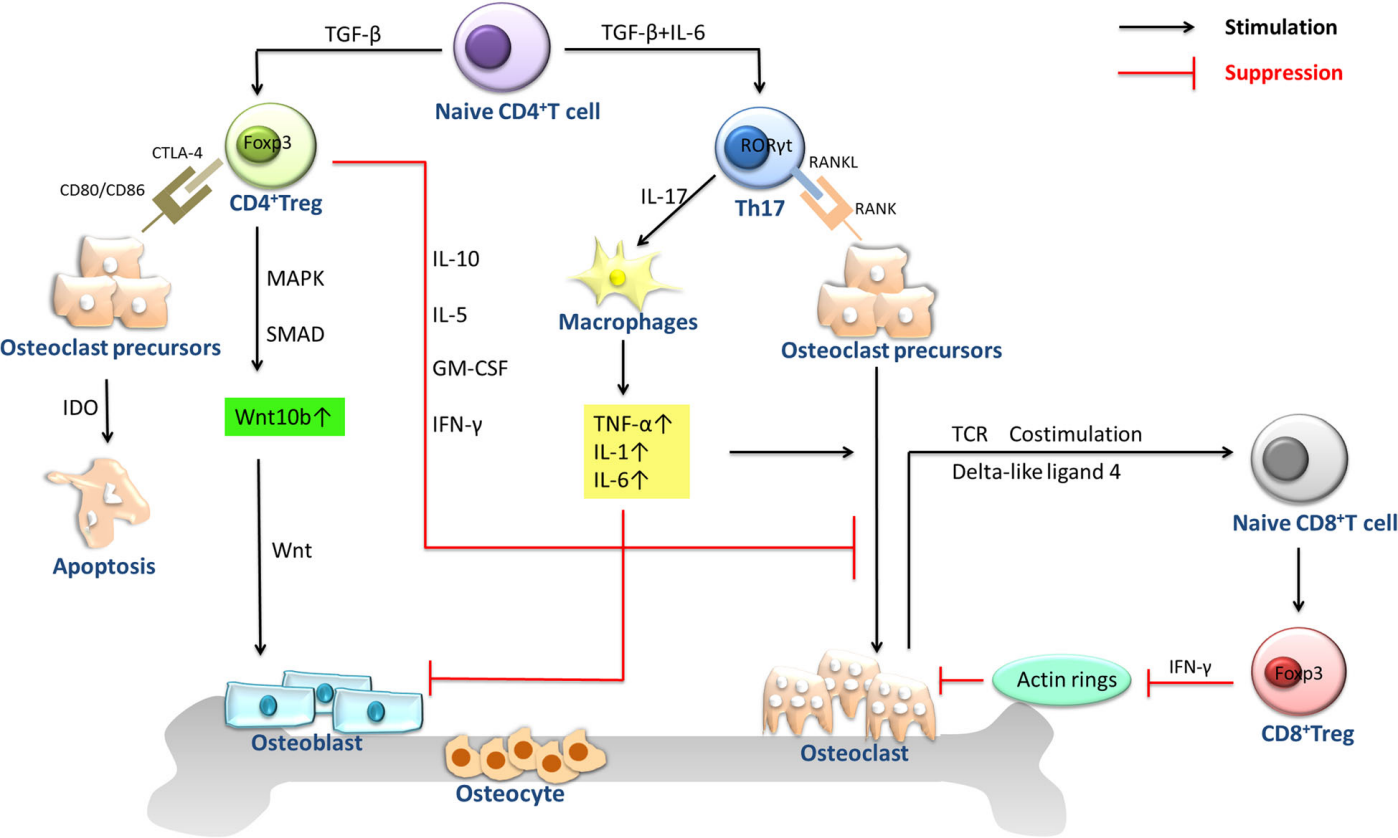
CD4^+^Treg cells affect the bone include cell contact-dependent mechanisms and inhibitory cytokine inhibition mechanisms. CD4^+^Treg cells can promote the proliferation and differentiation of osteoblasts by secreting TGF-β and activating intracellular effectors such as MAPK and Smad-related proteins that induce mesenchymal stem cells to differentiate into osteoblasts and promote the proliferation and differentiation of these osteoblasts. CD8^+^Treg cells can inhibit the maturation and activity of osteoclasts by suppressing the formation of their actin rings. Simultaneously, in the bone marrow, the unique property of osteoclasts to induce CD8^+^Treg cells and the ability of CD8^+^Treg cells to regulate osteoclast function established a bi-directional regulatory loop between the two types of cells. Th17 cells express high level of RANKL on its surface, which binds to RANK on the surface of osteoclast precursor cells, promoting the development of osteoclast precursor cells to osteoclasts to accelerate bone absorption. Th17 cells also can secrete IL-17 which directly enhances the expression of RANKL in osteoclastogenesis-supporting cells

Osteoporosis patients usually show an increase in bone turnover, which leads to an imbalance of bone resorption and bone formation [1]. Bone development is a process of dynamic balance that is achieved by bone remodelling. Bone remodelling is a process during which bone function constantly adapts to changes in mechanical and physiological stress. It can allow the shaping and repair of bone morphology [5, 6]. Osteoblasts and osteoclasts play a major role in bone remodelling, and any imbalance between them causes various metabolic bone diseases [5]. In recent years, many studies have confirmed that immune cells can interact with osteoblasts and osteoclasts to regulate bone formation and resorption and that macrophage colony-stimulating factor (M-CSF) and receptor activator of nuclear factor-kB ligand (RANKL) act as a bridge between the immune system and bone system [7]. Osteoclasts, originating from haematopoietic stem cells, are multinucleated cells formed after the fusion of precursor cells of the monocytic lineage. Induction of osteoclast formation requires M-CSF and RANKL [8]. In the process of bone resorption, RANKL activates the nuclear factor-kB receptor activator (RANK) receptor on the membrane surface of osteoclast precursor cells and osteoclasts, which leads to the formation and activation of osteoclasts, thus affecting bone remodelling [9]. M-CSF promotes the proliferation and survival of osteoclast precursor cells mainly by activating extracellular signal regulated kinase (ERK) via growth factor receptor binding protein 2(Grb2) and protein kinase B (Akt) via phosphoinositide 3 kinase (PI3K) [10]. T cells account for approximately 5% of bone marrow cells in the bone marrow stroma and parenchyma. T cells can differentiate into CD4^+^ T cells and CD8^+^ T cells. Naive CD4^+^ T cells can differentiate into Th1, Th2, Th9, Th17, Th22, and Treg cells and follicular helper T (Tfh) cells [3]. Th17 cells and Treg cells play important roles in maintaining bone homeostasis, especially in osteoclast differentiation (Fig. 1) [11].

### The relationship between Treg cells and bone loss

In 1995, Sakaguchi et al. first discovered Treg cells in the study of autoimmune diseases in mice [12]. Since then, Treg cells have become a hotspot of research on autoimmune diseases, tumours and other diseases. Treg cells mature in the thymus. Interleukin-2 (IL-2) plays an important role in the survival and development of Treg cells. Foxp3, a member of the forkhead box family of transcription factors, is currently recognized as a specific identification marker of Treg cells and is also an essential molecule for the development and functional expression of Treg cells [13]. Treg cells are mainly divided into two categories: naturally occurring Treg cells (nTregs) and induced Treg cells (iTregs). nTregs exist naturally in the thymus, and iTregs are generated from naive T cells in peripheral lymphoid tissues under stimulation by self-antigens [14]. M-CSF and RANKL, which induce the differentiation of osteoclasts are produced under the action of immune cells, bone marrow stromal cells, osteoblasts and fibroblasts [7]. Treg cells have immunosuppressive functions. They can inhibit the production of osteoclasts by preventing the production of RANKL and M-CSF, leading to an increase in bone mass [15]. Studies have shown that the main mechanisms through which Treg cells affect bone include cell contact-dependent mechanisms and inhibitory cytokine inhibition mechanisms [16]. Recently, it has been pointed out that nTregs mainly inhibit the production of osteoclasts through a cell contact-dependent mechanism, while the inhibitory effect of iTregs occurs through an inhibitory cytokine-dependent mechanism [6]. Cytotoxic T lymphocyte-associated antigen-4 (CTLA-4) is an important surface molecule involved in Treg cell-mediated cell contact-dependent inhibition of osteoclast generation [17]. Treg cells expressing CTLA-4 bind to CD80/CD86 on the surface of osteoclast precursor cells and induce the activation of indoleamine-2,3-dioxygenase in osteoclast precursor cells. Activated indoleamine-2,3-dioxygenase can degrade tryptophan, promote the apoptosis of osteoclast precursor cells, and thus inhibit bone resorption [18]. In addition to triggering immunosuppression through direct contact between cells, Treg cells can also secrete inhibitory cytokines that have indirect immunosuppressive activity. IL-10 is one of the inhibitory cytokines secreted by Treg cells and can inhibit the proliferation of T cells and the production of cytokines by T cells. IL-10 can inhibit the differentiation and maturation of osteoclasts by upregulating the secretion of osteoprotegerin (OPG) and downregulating the expression of RANKL and M-CSF [5, 19]. IL-35 is a newly discovered cytokine secreted by Treg cells that can reduce the expression of IL-17, thereby reducing the progression of collagen-induced arthritis in mice [20]. It has been demonstrated that after injection of mice with Treg cells that were amplified and purified in vitro with magnetic beads and coated with anti-CD3 and anti-CD28 antibodies, the expression of cytokines inhibiting osteoclast generation, such as granulocyte-macrophage colony-stimulating factor (GM-CSF), interferon-γ (IFN-γ), IL-5 and IL-10, increased significantly in the mice [17, 21]. In addition, evidence has shown that Treg cells also have certain effects on osteoblasts [22]. Treg cells can promote the proliferation and differentiation of osteoblasts by secreting TGF-β and activating intracellular effectors such as mitogen activated protein kinase (MAPK) and Smad-related proteins that induce mesenchymal stem cells to differentiate into osteoblasts and promote the proliferation and differentiation of these osteoblasts [23]. In addition, on the surface of osteoblasts, there are specific receptors for each subtype of TGF-β. Binding of TGF-β to its receptor on the surface of osteoblasts can accelerate the generation of osteoblasts through the Smad protein. The Smad protein has been shown to be directly involved in TGF-β signalling pathway-induced osteoblast formation [23, 24]. Wnt10b is an osteogenic Wnt ligand that can activate Wnt signalling in osteoblasts. Treg cells are involved in upregulation of Wnt10b by CD8^+^T cells during intermittent PTH treatment and supplementation with the *probiotic Lactobacillus rhamnosus* GG [14, 25]. Recently, the CD8 counterpart of Treg cells has been discovered and is called Foxp3^+^CD8^+^Treg cells [3]. These cells do not affect the survival of osteoclasts, but they can inhibit the maturation and activity of osteoclasts by suppressing the formation of their actin rings. Simultaneously, in the bone marrow, the unique property of osteoclasts to induce Foxp3^+^CD8^+^Treg cells and the ability of Foxp3^+^CD8^+^Treg cells to regulate osteoclast function establishes a bi-directional regulatory loop between these two types of cells [26]. Interestingly, this regulatory loop does not require the presence of various pro-inflammatory cytokines [26]. Unlike CD4^+^Treg cells which are present in large numbers in peripheral blood and the lymphatic circulation (accounting for approximately 5–12% of all CD4^+^T cells), CD8^+^Treg cells are present in small numbers in peripheral blood and the lymphatic circulation, accounting for only 0.2–2% of total CD8^+^T cells in various lymphoid organs [27]. Thus, current studies on CD8^+^Treg cells are not sufficient, and the role of CD8^+^Treg cells in osteoporosis has not yet been fully illustrated. Therefore, further studies in this area are needed [3]. However, it can be confirmed that a decrease in the number or function of CD4^+^Treg cells and CD8^+^Treg cells in the human body will cause an increase in bone loss and consequently lead to osteoporosis.

### The relationship between Th17 cells and bone loss

Immature T cells can differentiate into Th17 cells under stimulation by TGF-β and the inflammatory response. In addition, IL-6, IL-1β and IL-23 can affect the differentiation and development of Th17 cells [5]. Retinoic acid-related orphan receptor-γt (RORγt) is an important transcription factor of Th17 cells that is responsible for pathological immune responses. Th17 cells not only can secrete IL-17, IL-21 and IL-22, but also can produce IFN-γ [28]. Among these cytokines, IL-17 is the most important pro-inflammatory factor. The IL-17 family has six members: IL-17A-IL-17F [29]. Th17 cells control bone mass in two ways. On the one hand, Th17 cells express high surface levels of RANKL, which binds to RANK on the surface of osteoclast precursor cells, promoting the differentiation of osteoclast precursor cells into osteoclasts to accelerate bone absorption. On the other hand, Th17 cell-secreted IL-17 directly enhances the expression of RANKL in osteoclastogenesis-supporting cells such as osteoblasts and synovial fibroblasts [30]. RANKL binds to RANK on the surface of osteoclast precursor cells and promotes the maturation of osteoclasts, leading to an increase in bone resorption [7]. Moreover, IL-17 can also induce macrophages to produce a variety of inflammatory factors, such as TNF-α, IL-1 and IL-6, to activate and intensify the local inflammatory response, which indirectly promotes the expression of RANKL in osteoclastogenesis-supporting cells, enhances the binding of RANKL to RANK on the surface of osteoclast precursor cells, and synergistically accelerates bone absorption by osteoclasts [31]. A very important activity of IL-17 is that it triggers the production of high levels of RANKL by upregulating the production of RANK, which is crucial for the interaction between T lymphocytes and bone cells (Table 1). Therefore, in some studies, Th17 cells are called osteoclast subsets of T lymphocytes [32]. Many clinical analyses have shown that the number of Th17 cells in the blood and surrounding tissues of osteoporosis patients is several fold higher than that in the osteoporosis-free population. Thus, the Th17 cell count can be used as an important marker for osteoporosis [3]. Studies have demonstrated that after ovariectomy (OVX), the level of IL-17 is significantly increased in rats. An anti-IL-17 antibody antagonist was found to effectively prevent bone damage caused by oestrogen reduction, indicating that IL-17 is involved in bone resorption [33].

**Table 1:** Role of Treg cells and Th17 cells cytokines in osteoimmune system

Cytokine	Source	Effect on bone mass	Function in bone homeostasis	References
IL-1	Macrophages	↓	Enhances the expression of RANKL to promote osteoclastogenesis	[31]
IL-5	Th2	↑	Inhibits osteoclastogenesis	[21]
IL-6	Macrophages	↓	Activates osteoclastogenesis	[31]
IL-10	Treg	↑	Inhibits bone resorption	[5, 19]
IL-17	CD4^+^ T cells	↓	Enhances the expression of RANKL and induces macrophages to produce a variety of inflammatory factors	[28, 30, 31]
IL-35	Treg	↑	Reduces the expression of IL-17 Inhibits osteoclastogenesis	[20]
RANK	Osteoclasts	↓	Osteoclast differentiation and activation	[9]
RANKL	Th17	↓	Osteoclast activation through RANK	[7]
GM-CSF	Th1	↑	Inhibits osteoclastogenesis	[17]
IFN-γ	Activated Th cells, NK cells	↑	Inhibits osteoclastogenesis	[17]
TGF-β	Multiple cells lines	uncertain	Activates osteoclast Induces osteoblast formation	[5, 23, 24]
TNF-α	Th17, macrophages	↓	Activates osteoclastogenesis through RANKL	[31]

### The correlation between Treg cells and Th17 cells

CD4^+^ T cells are the common precursor cells of Treg cells and Th17 cells. Differentiation of Treg cells and Th17 cells requires the involvement of the TGF-β- regulated signalling pathway [34]. However, in different cytokine environments, the differentiation direction of CD4^+^ T cells can be changed. In the presence of IL-6, IL-23 and TGF-β, IL-6 can inhibit the expression of Foxp3 by activating signal transducer and activator of transcription 3 (STAT3) and can upregulate IL-23 receptor expression to induce immature T cells to differentiate into Th17 cells. In contrast, in the absence of IL-6 and other pro-inflammatory factors, TGF-β drives the differentiation of immature T cells into Treg cells [35]. It has also been reported that in human T cells cultured in vitro, the absence of IL-6, IL-21 and TGF-β can induce RORγt production, upregulate IL-23 receptor expression, inhibit Foxp3 expression, and promote the differentiation of Th17 cells. In addition, Th17 cells can secrete IL-21 to further promote the generation of Th17 cells [36].. Retinoic acid is a key regulator of the TGF-β-dependent immune response. It can inhibit RORγt and promote Treg cell differentiation under the inductive effects of Th17 cells [37]. Recent studies have identified a new subset of Treg cells called CD39^+^Foxp3^+^Treg cells. These cells can inhibit the secretion of IL-17 by Th17 cells, thereby inhibiting autoimmune inflammation induced by IL-17 [38]. Interestingly, Th17 cells and Treg cells can also interconvert. For example, when the concentration of cytokines produced by exogenous Th17 cells increases, Treg cells are transformed into IL-17- secreting cells [39]. Yang et al. found that in the presence of IL-6 and TGF-β or IL-1 and IL-23, both nTregs and iTregs can be transformed into Th17 cells [40]. Foxp3/ IL-17 double-positive T cells act as intermediate cells in the transformation of Th17 cells into Treg cells [41]. Because Th17 cells and Treg cells are associated with each other, there is a balance between Th17 cells and Treg cells when they function in the human body [42]. Considering the effects of Treg cells and Th17 cells on bone loss, we may conclude that Th17 cells can promote bone resorption while Treg cells can inhibit bone resorption [43]. Therefore, by regulating the cross-talk between the Th17/Treg cell balance and bone cells, we may find new approaches for the treatment of osteoporosis.

### Factors affecting the balance between Th17 cells and Treg cells

#### Signalling pathways

The signalling pathways involved in the Th17/Treg cell balance include the Notch signalling pathway, T cell receptor (TCR) signalling pathway and costimulatory molecule signalling pathway.

The Notch signalling pathway is a highly conserved intercellular communication cascade in multicellular organisms that can regulate the fate of various cells and differentiation processes in the human immune system. The Notch pathway includes four Notch receptors (Notch1, Notch2, Notch3 and Notch4) and five ligands (Jagged1, Jagged2, Delta-like1, Delta-like3 and Delta-like4) [44]. Li et al. showed that Notch1 mRNA expression was positively correlated with the Th17/Treg ratio. In the inflammatory response, when Notch1 signalling was enhanced, the expression of RORγt was significantly increased but the expression of Foxp3 was significantly decreased, thereby regulating the differentiation of Th17 cells and Treg cells [45]. Yin et al. found that blocking Notch signalling with DAPT (a γ-secretase inhibitor) significantly inhibited the differentiation of Th17 cells and reduced the number of Th17 lineage cells, leading to a reduction in IL-17 secretion, which suggests that inactivation of Notch signalling may reduce the production of IL-17 [46]. Notch signalling molecules can regulate the Th17/Treg cell balance by inducing the transformation of immature CD4^+^ T cells into Th17 cells and Treg cells: Jagged1 reduces the expression of IL-6 and TGF-β-induced RORγt in CD4^+^ T cells, inhibiting the conversion of CD4^+^ T cells into Th17 cells. In addition, Jagged1 and 2, together with Delta-like1 and 4, can enhance the conversion of CD4^+^ T cells into Treg cells by regulating the TGF-β signalling pathway and Foxp3 [47]. Although the mechanism of the Notch signalling pathway in osteoporosis is not yet completely understood, we can still see that regulating the Th17/Treg cell balance by reducing the differentiation and function of Th17 cells by inhibiting the activity of the Notch signalling pathway might be a potential therapeutic approach for the treatment of osteoporosis [48].

The TCR signalling pathway also has some influence on the growth and development of Treg cells [49]. When the key enzymes in TCR stimulation-induced signal cascade reactions, such as lymphocyte protein tyrosine kinase (LCK), ζ chain related protein kinase (Zap70) and the adaptor used to activate T cells (LAT) contain mutations or deletions, the TCR signal is weakened, which leads to the development of defects and reductions in the activity of Treg cells, and simultaneously stimulates the production of IL-6 to drive the differentiation of CD4^+^ T cells into Th17 cells. However, when all components of the TCR signalling pathway are normal, inhibition of TCR signalling promotes the generation of Treg cells [50–52]. For example, a CD3ζ mutant with a phosphorylation defect can weaken TCR signalling but promote Treg cell generation [53]. Interestingly, TCR signalling mainly affects nTregs. Whether it has any effect on the differentiation of iTregs remains to be studied [49]. Some scholars claim that the levels of IL-17 and Foxp3 do not increase when the Src family kinase LCK is mutated, indicating that the numbers of Th17 cells and Treg cells do not change, a possibility that is worthy of further study [54]. Treg cells can further differentiate into effector Treg cells after activation of the TCR signalling pathway and exhibit an activated phenotype and full suppressor function. Interferon regulatory factor 4 (IRF4) plays a synergistic role in this process by driving the expression of the immunosuppressive cytokine IL-10 [55]. Bach2 is an important regulator of the maintenance of the stable state of downstream TCR signalling and the differentiation of Treg cells. It can limit the production of IL-10 and prevent the premature differentiation of Treg cells. Bach2 can inhibit the genomic-binding of IRF4, thus limiting the effector differentiation of Treg cells driven by TCR. Bach2 balances the transcriptional activity of IRF4 induced by TCR signalling to maintain homeostasis of nTregs and iTregs [56]. In addition, casein kinase 2 (CK2), as an enzyme modifying the TCR signalling pathway, plays an important role in the regulation of the Th17/Treg cell balance. Recently, a study reported that CK2 can promote Th17 cell differentiation and inhibit Treg cell generation by inhibiting FoxO1. If FoxO1 is knocked out or chemically inhibited, the number of Th17 cells is significantly decreased while the number of Treg cells is increased [57].

Activation of T cells requires the participation of a dual signal system. In addition to the first signal provided by TCR recognition of MHC-restricted antigenic peptide epitopes, the second signal provided by costimulatory molecules on antigen- presenting cells (APCs) is also needed to activate T cells [58]. The costimulatory molecules CD80 and CD86 on the surface of APCs bind to CD28 on the surface of T cells. The cytoplasmic tail of CD28 has docking sites for signalling molecules, among which the YMNM motif at the membrane-proximal end binds to PI3K, and the PYAP motif at the distal end binds to growth factor receptor binding protein 2 (Grb2) and Lck [59]. CD28 signal transduction is important to maintain the stability and function of Treg cells. Costimulatory signals are transmitted to developing thymocytes through the Lck binding motif in the cytoplasmic tail of CD28, thus inducing Foxp3 expression and upregulating the expression of glucocorticoid-induced tumour necrosis factor receptor (GITR) and CTLA-4 to initiate the differentiation of Treg cells [60]. In CD28-deficient mice, the number of nTregs and iTregs is decreased [61]. In addition, the CD28 costimulatory signal can also enhance the secretion of IFN-γ and IL-2 from activated CD4^+^ T cells. IL-2 can inhibit the expression of the α-chain of the IL-6 receptor, and IFN-γ inhibits STAT3 and further blocks the activation of Th17 cells by IL-6. These events form a negative regulatory loop modulating the differentiation of Th17 cells [62, 63].

### Metabolism

Nutrient metabolism in the human body is also important for maintenance of the Th17/Treg cell balance [64]. The energy demand of immature T cells is low. The ATP required for T cell activity is mainly produced by aerobic oxidation of glucose or by fatty acid oxidation. When T cells are activated, the glycolysis pathway becomes the main energy source due to active cell proliferation and growth [37]. The mammalian target of rapamycin (mTOR) protein regulates the key factor in T cell differentiation and function. Under steady-state conditions, mTOR is inhibited. When immature T cells recognize antigens, mTOR is activated and promotes the differentiation of T cells into different cell subtypes [37, 65]. Cluxton et al. demonstrated that the differentiation of Th17 cells mainly depends on glycolysis and hypoxia inducible factor-1 α (HIF-1α) because when the glucose level in mice was reduced or the mTOR inhibitor rapamycin was used, the number of Th17 cells decreased but the number of Treg cells increased in these mice. Treg cells also depend on glycolysis to some extent, but they are less dependent on glycolysis than Th17 cells. The differentiation of Treg cells requires oxidative phosphorylation and can be inhibited by HIF-1α [66]. Cluxton also pointed out that Treg cells can exhibit enhanced glycolysis, mitochondrial respiration and fatty acid oxidation, but Th17 cells appear dependent on fatty acid synthesis [66].

### Diet

Diet is closely correlated with human health. Excessive salt intake is not conducive to human health because a high-salt diet can cause a series of diseases, such as hypertension and diabetes [67]. Hamid Y. Dar et al. pointed out that excessive salt intake can lead to increased bone loss because a high-salt diet increases the expression of pro-inflammatory factors such as IL-6, IL-17, RANKL and TNF-α and decreases the expression of anti-inflammatory factors such as IL-10 and IFN-γ, which subsequently enhances the induction of Th17 cells and simultaneously decreases the number of Treg cells [68]. Yang et al. showed that high-salt diet can drive thymic Treg cells to adopt a Th17-like phenotype and promote the production of induced Treg cells with a Th17-like phenotype in a serum/glucocorticoid-regulated kinase 1 (SGK1) dependent manner, while maintaining their inhibitory function. SGK1 is a salt receptor in T cells and is preferentially translated in activated Treg cells. High- salt-induced activation of SGK1 signalling can directly promote the expression of RORγt in Foxp3^+^Treg cells, thereby playing an upstream role in Th17 polarization [69]. L. Wu et al. concluded that increased bone resorption after high-sodium diet intake not only may be a secondary cause of urinary calcium loss, but also may be due to a direct cell-mediated effect on osteoclasts. In their experiment, they found that higher concentrations of Na^+^ can significantly increase the expression of some transcription factors for osteoclastogenesis, such as nuclear factor-activated T cells c1 (NFATc1) and spleen proviral integration oncogene (SPI1). Importantly, NFATc1 is considered to be the most potent transcription factor induced by RANKL [70]. Interestingly, Agnes Schroder and colleagues found that a low-salt diet (LSD) increased bone density, reduced the number of osteoclasts, and increased the Na^+^ content and nuclear factor of activated T cell 5 (NFAT5) levels in bone marrow compared with those in mice on a high-salt diet. Mechanistically, local Na^+^ accumulation in the bone marrow of LSD-treated mice increased the expression of OPG and prevented RANKL-induced osteoclast formation in an NFAT5-dependent manner [71]. In addition, MacGregor and Lin et al. demonstrated that a reduction in salt intake may have an important beneficial effect on bone density, thus preventing and treating osteoporosis [72, 73]. En-De Hu et al. showed that the Treg/Th17 cell ratio in mice fed a high-fibre diet and sodium butyrate was significantly higher than that in mice in the model control group. A high-fibre diet and sodium butyrate can reduce the mRNA expression of IL-17 and IL-6, and increase the expression of IL-10 and TGF-β [74]. A high-fibre diet can induce the production of short-chain fatty acids (SCFAs) such as butyrate and propionate. In a mouse model of inflammatory bowel disease, administration of SCFAs was found to increase the level of Treg cells in the intestine, especially in the colon, via certain G-protein-coupled receptors or via inhibition of histone deacetylases [75]. Some scholars believe that SCFAs can act on the free fatty acid receptors GPR43, GPR41 and GPR109A to exert their effects on host immunity [76]. GPR43 expression is essential for the expansion and inhibition of Treg cells in colitis induced by SCFAs [77]. GPR109A is a receptor that responds to both niacin and butyrate [76]. Activation of GPR109A by SCFAs can upregulate the expression of anti-inflammatory molecules in monocytes, increase the differentiation of Treg cells and enhance the production of IL-10 [78]*.* Vitamin A is a fat-soluble vitamin and retinoic acid is its biologically active form. Vitamin A is highly concentrated in the intestine and is the core mediator of Treg cell homeostasis in the intestine. In the presence of TGF-β1, retinoic acid can induce the differentiation of Treg cells. Retinoic acid can not only enhance Treg cell differentiation but also prevent Th17 cell differentiation [79, 80]. Interestingly, SCFAs may also stimulate the production of retinoic acid by epithelial cells [81]. Mice fed a diet lacking vitamin A or treated with retinoic acid receptor inhibitors show a reduction in the population of Treg cells [82, 83]. In addition, dietary amaranth can reduce the internal level of IL-17 while increasing the level of IL-10 and can reduce the Th17/Treg cell ratio to provide immunomodulatory effects through its abundant beneficial compounds [84].

### The intestinal microflora

**Fig. 2:**
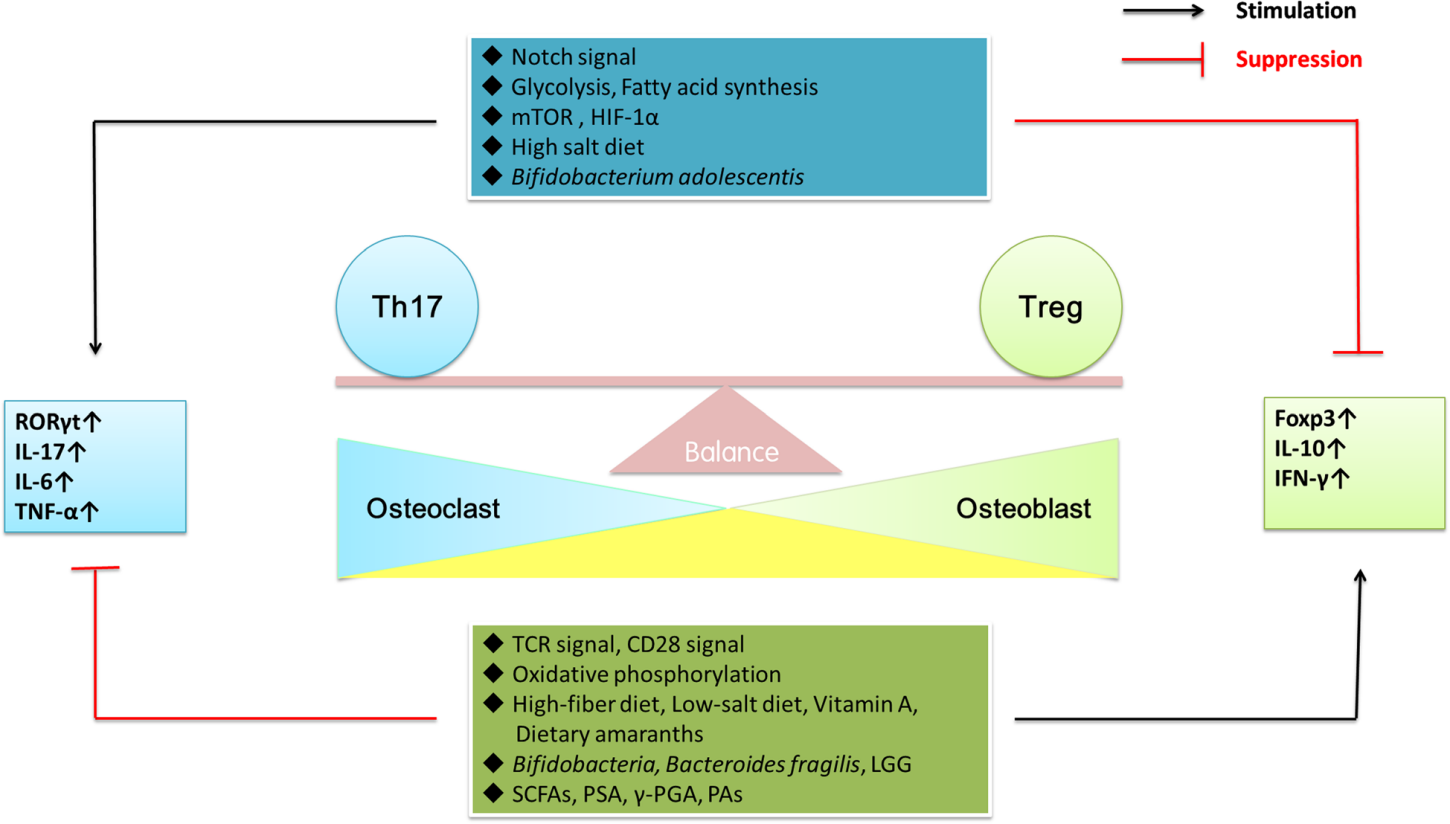
There are many factors that can affect the Th17/Treg cell balance include Notch signalling pathway, TCR signalling pathway, costimulatory signalling pathway, metabolism, diet and the intestinal microflora

The intestinal microflora is not only involved in the regulation of various physiological functions in the human body but also related to many human diseases. Importantly, the intestinal microflora may be a key regulatory factor of bone metabolic homeostasis [85]. The intestinal microflora mainly consists of five different phyla and several genera of the Eubacteria domain, including Actinobacteria (*Bifidobacterium*), Bacteroidetes (*Bacteroides*), Firmicutes (*Lactobacillus*), Proteobacteria (*Escherichia*), and Verrucomicrobia (*Akkermansia*) [86]. *Bifidobacteria* can promote monocytes to secrete large amounts of TGF-β to induce Treg cell differentiation [87]. Interestingly, the human symbiotic species *Bifidobacterium adolescentis*, can independently induce the production of Th17 cells in the intestines of mice [88]. Sarah Onuora et al. found that *Bifidobacterium adolescentis* worsened autoimmune arthritis in a mouse model [89]. The role of *Bacteroides fragilis* is largely dependent on polysaccharide A (PSA), an immunomodulator that maintains host immune homeostasis. PSA can promote the differentiation of CD4^+^T cells into Treg cells. In addition, it can inhibit the differentiation of Th17 cells through Toll-like receptor signalling inherent in CD4^+^T cells [90]. Hamid Y. Dar et al. found that oral administration of *Bacillus clausii* in mice with postmenopausal osteoporosis reduced the levels of pro-inflammatory cytokines (IL-6, IL-17 and TNF-α) and increased the levels of anti-inflammatory cytokines (IL-10 and IFN-γ), thereby enhancing bone health [91]. You Jin Jang and colleagues isolated novel strains of *Lactobacillus fermentum* (KBL374 and KBL375) from faeces. When they used these two strains to treat human peripheral blood mononuclear cells, they found that the levels of inflammatory cytokines such as IL-17A were decreased but those of anti-inflammatory cytokines such as IL-10 were increased. Administration of *Lactobacillus fermentum* KBL374 or KBL375 to mice increased the population of CD4 ^+^ CD25 ^+^ Foxp3 ^+^ Treg cells in mesenteric lymph nodes [92]. Abdul Malik Tyagi and his team treated neonatal mice with *Lactobacillus rhamnosus* GG (LGG) and found that the trabecular bone volume in treated mice was increased. Mechanistically, butyrate produced by LGG in the intestine may induce the expansion of Treg cells. Treg cells promote the assembly of the NFAT1-SMAD3 transcription complex in CD8^+^ cells. NFAT1-SMAD3 drives the expression of Wnt10b, which consequently regulates bone anabolism [25]. In addition to producing SCFAs and PSA, the intestinal microflora may also produce the aryl hydrocarbon receptor (AHR), polyamines (PAs) and poly-gamma-glutamic acid (γ-PGA) [78]. AHR regulates the differentiation of Treg cells and Th17 cells in a ligand-specific manner. For example, when activated by TCDD (2,3,7, 8-tetrachlorodibenzo-p-dioxin), AHR can induce the generation of Treg cells and suppress experimental autoimmune encephalomyelitis (EAE) through a TGF- β1-dependent mechanism. In contrast, after FICA (6-formylindolo [3,2-b] carbazole) treatment, AHR can promote Th17 cell differentiation and exacerbate EAE [93]. Recently, a team proposed that AHR binds directly to the open chromatin regions in the locus of the orphan chemoattractant receptor GPR15 to enhance its expression and thus regulates intestinal homing of Treg cells [94]. PAs are small polycationic molecules produced during arginine metabolism. Spermidine is the best characterized PA to date. Carriche and his colleagues found that spermidine enhanced Treg cell differentiation in vitro in an autophagy-related manner [95]. γ-PGA can induce the expression of Foxp3 through the Toll-like receptor 4 pathway, thus promoting Treg cell differentiation. γ-PGA can also inhibit the differentiation of Th17 cells by suppressing the expression of IL-6 [96]. In summary, the microflora plays an important role in regulating the maintenance and function of intestinal Treg cells and Th17 cells, although the mechanisms through which the microflora regulates the balance between Th17 cells and Treg cells are not yet fully understood (Fig. 2) [97].

## Conclusion

In conclusion, the impact of the balance between Th17 cells and Treg cells on bone mass is obvious. If the Th17/Treg cell balance shifts towards Th17 cells, bone resorption is enhanced, and the risk of osteoporosis is greatly increased. Currently, the treatment of osteoporosis mainly includes oestrogen replacement, phosphate treatment, calcium and vitamin D treatment, and appropriate physical activities. Considering the close correlation between Th17 cells and Treg cells and their plasticity, we believe that there are other influencing factors in addition to signalling pathways, metabolism, diet and the intestinal microflora. In-depth study of the factors that affect Th17/Treg cell balance in osteoporosis will help to further identify targets for new osteoporosis drugs, which are also crucial for the maintenance of human health. The Th17/Treg cell balance also has a profound impact on the treatment of cancer and autoimmune diseases. However, most of the current studies are carried out in animal models. In the future, more high-quality clinical studies are needed to further explore the effectiveness and safety of regulating the Th17/Treg cell balance in the treatment of osteoporosis.

## Data Availability

Not applicable.

## Abbreviations

M-CSF: Macrophage colony-stimulating factor
RANKL: Receptor activator of nuclear factor-kB ligand
RANK: Nuclear factor-kB receptor activator
ERK: Extracellular signal regulated kinase
Akt: Protein kinase B
Grb2: Growth factor receptor binding protein 2
PI3K: Phosphoinositide 3 kinase
Tfh: Follicular helper T cells
IL-2: Interleukin-2
nTregs: Naturally occurring Tregs cells
iTregs: Induced Tregs cells
CTLA-4: Cytotoxic T lymphocyte-associated antigen-4
OPG: Osteoprotegerin
GM-CSF: Granulocyte macrophage colony stimulating factor
IFN-γ: Interferon-γ
RORγt: Retinoic acid-related orphan receptors-γt
OVX: Ovariectomy
STAT3: Signal transducer and activator of transcription 3
TCR: T cell receptor
LCK: Lymphocyte protein tyrosine kinase
Zap70: ζ chain related protein kinase
LAT: Adaptor used to activate T cells
CK2: Casein kinase 2
APC: Antigen presenting cell
Grb2: Growth factor receptor binding protein 2
GITR: Glucocorticoid-induced tumor necrosis factor receptor
mTOR: The target protein of rapamycin
HIF-1α: Hypoxia inducible factor-1 α

## References

[1] Compston JE, McClung MR, Leslie WD (2019). Osteoporosis. Lancet.

[2] Dar H, Azam Z, Anupam R, Mondal R, Srivastava R (2018). Osteoimmunology: the Nexus between bone and immune system. Front Biosci.

[3] Srivastava RK, Dar HY, Mishra PK (2018). Immunoporosis: immunology of osteoporosis-role of T cells. Front Immunol.

[4] Arron JR, Choi Y (2000). Bone versus immune system. Nature..

[5] Dar HY, Azam Z, Anupam R, Mondal RK, Srivastava RK (2018). Osteoimmunology: The Nexus between bone and immune system. Front Biosci.

[6] Bozec A, Zaiss MM (2017). T regulatory cells in bone Remodelling. Curr Osteoporos Rep.

[7] Takayanagi H (2007). Osteoimmunology: shared mechanisms and crosstalk between the immune and bone systems. Nat Rev Immunol.

[8] Takayanagi H (2010). The unexpected link between osteoclasts and the immune system. Oxygen Transpor Tissue XXXIII.

[9] Takayanagi H, Sato K, Takaoka A, Taniguchi T (2005). Interplay between interferon and other cytokine systems in bone metabolism. Immunol Rev.

[10] Ross FP, Teitelbaum SL (2006). alphavbeta3 and macrophage colony-stimulating factor: partners in osteoclast biology. Immunol Rev.

[11] Vries T, Bakkali I, Kamradt T, Schett G, Jansen I, d'amelio P. What Are the Peripheral Blood Determinants for Increased Osteoclast Formation in the Various Inflammatory Diseases Associated With Bone Loss? Front Immunol. 2019:10. 10.3389/fimmu.2019.00505 PMC6434996 30941138

[12] Sakaguchi S. Immunologic self-tolerance maintained by activated T cells expressing IL-2 receptor alpha-chains (CD25). Breakdown of a single mechanism of self-tolerance causes various autoimmune diseases. J Immunol (Baltimore, Md : 1950). 1995;3(155):1154–64. 7636184

[13] Shao TY, Hsu LH, Chien CH, Chiang BL (2016). Novel Foxp3(−) IL-10(−) regulatory T-cells induced by B-cells alleviate intestinal inflammation in vivo. Sci Rep.

[14] Yu M, D'Amelio P, Tyagi AM, Vaccaro C, Li JY, Hsu E (2018). Regulatory T cells are expanded by Teriparatide treatment in humans and mediate intermittent PTH-induced bone anabolism in mice. EMBO Rep.

[15] Okamoto K, Nakashima T, Shinohara M, Negishi-Koga T, Komatsu N, Terashima A (2017). Osteoimmunology: the conceptual framework unifying the immune and skeletal systems. Physiol Rev.

[16] Zaiss MM, Axmann R, Zwerina J, Polzer K, Guckel E, Skapenko A (2007). Treg cells suppress osteoclast formation: a new link between the immune system and bone. Arthritis Rheum.

[17] Yuan FL, Li X, Lu WG, Xu RS, Zhao YQ, Li CW (2010). Regulatory T cells as a potent target for controlling bone loss. Biochem Biophys Res Commun.

[18] Fischer L, Herkner C, Kitte R, Dohnke S, Riewaldt J, Kretschmer K, et al. Foxp3+ Regulatory T Cells in Bone and Hematopoietic Homeostasis. Front Endocrinol. 2019;10(578). 10.3389/fendo.2019.00578 PMC6746882 31551927

[19] Taylor A, Verhagen J, Blaser K, Akdis M, Akdis CA (2006). Mechanisms of immune suppression by interleukin-10 and transforming growth factor-β: the role of T regulatory cells. Immunology..

[20] Oh S, Rankin AL, Caton AJ (2010). CD4+CD25+ regulatory T cells in autoimmune arthritis. Immunol Rev.

[21] Kelchtermans H, Geboes L, Mitera T, Huskens D, Leclercq G, Matthys P. Activated CD4+CD25+ regulatory T cells inhibit osteoclastogenesis and collagen-induced arthritis. Ann Rheumatic Dis. 68(5):744–50. 10.1136/ard.2007.086066 18480308

[22] Tanaka Y (2019). Clinical immunity in bone and joints. J Bone Miner Metab.

[23] Runyan CE, Liu Z, Schnaper HW. Phosphatidylinositol-3-kinase and Rab5 inversely regulate the Smad anchor for receptor activation (SARA) protein independently of TGF-[beta]1. J Biol Chem. 2012. 10.1074/jbc.M112.380493 PMC3476251 22942286

[24] Zhao L, Jiang S, Hantash B (2009). Transforming growth factor β1 induces Osteogenic differentiation of murine bone marrow stromal cells. Tissue Eng A.

[25] Tyagi AM, Yu M, Darby TM, Vaccaro C, Li JY, Owens JA (2018). The microbial metabolite butyrate stimulates bone formation via T regulatory cell-mediated regulation of WNT10B expression. Immunity..

[26] Shashkova EV, Trivedi J, Cline-Smith AB, Ferris C, Buchwald ZS, Gibbs J (2016). Osteoclast-primed Foxp3<sup>+</sup> CD8 T cells induce T-bet, Eomesodermin, and IFN-γ to regulate bone Resorption. J Immunol.

[27] Niederkorn JY (2008). Emerging concepts in CD8+ T regulatory cells. Curr Opin Immunol.

[28] You L, Chen L, Pan L, Peng Y, Chen J (2018). SOST gene inhibits Osteogenesis from adipose-derived Mesenchymal stem cells by inducing Th17 cell differentiation. Cell Physiol Biochem.

[29] Li Q, Wang B, Mu K, Zhang JA (2019). The pathogenesis of thyroid autoimmune diseases: new T lymphocytes - cytokines circuits beyond the Th1-Th2 paradigm. J Cell Physiol.

[30] Ono T, Takayanagi H (2017). Osteoimmunology in bone fracture healing. Curr Osteoporos Rep..

[31] Raphael I, Nalawade S, Eagar TN, Forsthuber TG. T cell subsets and their signature cytokines in autoimmune and inflammatory diseases. Cytokine. 2014;74(1):5–17. 10.1016/j.cyto.2014.09.011 PMC4416069 25458968

[32] Xiong J, Piemontese M, Thostenson JD, Weinstein RS, Manolagas SC, O'Brien CA (2014). Osteocyte-derived RANKL is a critical mediator of the increased bone resorption caused by dietary calcium deficiency. Bone..

[33] Bandyopadhyay S, Lion J-M, Mentaverri R, Ricupero DA, Kamel S, Romero JR, et al. Attenuation of osteoclastogenesis and osteoclast function by apigenin. Biochem Pharmacol. 2006;72(2):184–97. 10.1016/j.bcp.2006.04.018 16750176

[34] Oukka M (2007). Interplay between pathogenic Th17 and regulatory T cells. Ann Rheum Dis.

[35] Bettelli E, Carrier Y, Gao W, Korn T, Strom TB, Oukka M (2006). Reciprocal developmental pathways for the generation of pathogenic effector TH17 and regulatory T cells. Nature..

[36] Kyburz D, Corr M. Th17 cells generated in the absence of TGF-β induce experimental allergic encephalitis upon adoptive transfer. Exp Rev Clin Immunol. 2011;7(3):283–5. 10.1586/eci.11.7 21595594

[37] Sun L, Fu J, Zhou Y (2017). Metabolism controls the balance of Th17/T-regulatory cells. Front Immunol.

[38] Fletcher JM, Lonergan R, Costelloe L, Kinsella K, Moran B, O'Farrelly C (2009). CD39+Foxp3+ regulatory T cells suppress pathogenic Th17 cells and are impaired in multiple sclerosis. J Immunol.

[39] Ueno A, Ghosh A, Hung D, Li J, Jijon H (2015). Th17 plasticity and its changes associated with inflammatory bowel disease. World J Gastroenterol.

[40] Yang XO, Nurieva R, Martinez GJ, Kang HS, Chung Y, Pappu BP, et al. Molecular Antagonism and Plasticity of Regulatory and Inflammatory T Cell Programs. Immunity. 2008;29(1):44–56. 10.1016/j.immuni.2008.05.007 PMC2630532 18585065

[41] Deknuydt F, Bioley G, Valmori D, Ayyoub M (2009). IL-1beta and IL-2 convert human Treg into T(H)17 cells. Clin Immunol.

[42] Knochelmann HM, Dwyer CJ, Bailey SR, Amaya SM, Elston DM, Mazza-McCrann JM (2018). When worlds collide: Th17 and Treg cells in cancer and autoimmunity. Cell Mol Immunol.

[43] Gruber R (2019). Osteoimmunology: inflammatory osteolysis and regeneration of the alveolar bone. J Clin Periodontol.

[44] Jiao WE, Wei JF, Kong YG, Xu Y, Tao ZZ, Chen SM (2019). Notch signaling promotes development of allergic rhinitis by suppressing Foxp3 expression and Treg cell differentiation. Int Arch Allergy Immunol.

[45] Li C, Sheng A, Jia X, Zeng Z, Zhang X, Zhao W (2018). Th17/Treg dysregulation in allergic asthmatic children is associated with elevated notch expression. J Asthma.

[46] Yin X, Wei H, Wu S, Wang Z, Liu B, Guo L (2019). DAPT reverses the Th17/Treg imbalance in experimental autoimmune uveitis in vitro via inhibiting notch signaling pathway. Int Immunopharmacol.

[47] Qin L, Zhou YC, Wu HJ, Zhuo Y, Wang YP, Si CY (2017). Notch signaling modulates the balance of regulatory T cells and T helper 17 cells in patients with chronic hepatitis C. DNA Cell Biol.

[48] Mijailovic I, Nikolic N, Djinic A, Carkic J, Milinkovic I, Peric M (2020). The down-regulation of notch 1 signaling contributes to the severity of bone loss in aggressive periodontitis. J Periodontol.

[49] Li MO, Rudensky AY (2016). T cell receptor signalling in the control of regulatory T cell differentiation and function. Nat Rev Immunol..

[50] Brisslert M, Bian L, Svensson MN, Santos RF, Jonsson IM, Barsukov I (2014). S100A4 regulates the Src-tyrosine kinase dependent differentiation of Th17 cells in rheumatoid arthritis. Biochim Biophys Acta.

[51] Picard C, Dogniaux S, Chemin K, Maciorowski Z, Lim A, Mazerolles F (2009). Hypomorphic mutation of ZAP70 in human results in a late onset immunodeficiency and no autoimmunity. Eur J Immunol.

[52] Cibrian D, Castillo-Gonzalez R, Fernandez-Gallego N, de la Fuente H, Jorge I, Saiz ML (2020). Targeting L-type amino acid transporter 1 in innate and adaptive T cells efficiently controls skin inflammation. J Allergy Clin Immunol.

[53] Hwang S, Song K-D, Lesourne R, Lee J, Pinkhasov J, Li L, et al. Reduced TCR signaling potential impairs negative selection but does not result in autoimmune disease. J Exp Med. 2012;209(10):1781–95. 10.1084/jem.20120058 PMC3457736 22945921

[54] Kemp KL, Levin SD, Stein PL (2010). Lck regulates IL-10 expression in memory-like Th1 cells. Eur J Immunol.

[55] Cretney E, Xin A, Shi W, Minnich M, Masson F, Miasari M (2011). The transcription factors Blimp-1 and IRF4 jointly control the differentiation and function of effector regulatory T cells. Nat Immunol.

[56] Sidwell T, Liao Y, Garnham AL, Vasanthakumar A, Gloury R, Blume J (2020). Attenuation of TCR-induced transcription by Bach2 controls regulatory T cell differentiation and homeostasis. Nat Commun.

[57] Gibson SA, Yang W, Yan Z, Qin H, Benveniste EN (2018). CK2 controls Th17 and regulatory T cell differentiation through inhibition of FoxO1. J Immunol.

[58] Salomon B, Lenschow DJ, Rhee L, Ashourian N, Singh B, Sharpe A (2000). B7/CD28 Costimulation is essential for the homeostasis of the CD4+CD25+ Immunoregulatory T cells that control autoimmune diabetes. Immunity..

[59] Esensten JH, Helou YA, Chopra G, Weiss A, Bluestone JA. CD28 Costimulation: From Mechanism to Therapy. Immunity. 44(5):973–88. 10.1016/j.immuni.2016.04.020 PMC4932896 27192564

[60] Tai X, Cowan M, Feigenbaum L, Singer A (2005). CD28 costimulation of developing thymocytes induces Foxp3 expression and regulatory T cell differentiation independently of interleukin 2. Nat Immunol.

[61] Zhang R, Huynh A, Whitcher G, Chang J, Maltzman JS, Turka LA (2013). An obligate cell-intrinsic function for CD28 in Tregs. J Clin Invest.

[62] Bouguermouh S, Fortin G, Baba N, Rubio M, Sarfati M (2009). CD28 co-stimulation down regulates Th17 development. PLoS One.

[63] Liao W, Lin J-X, Wang L, Li P, Leonard WJ. Modulation of cytokine receptors by IL-2 broadly regulates differentiation into helper T cell lineages. Nat Immunol. 2011;12(6):551–9. 10.1038/ni.2030 PMC3304099 21516110

[64] MacIver NJ, Michalek RD, Rathmell JC (2013). Metabolic regulation of T lymphocytes. Annu Rev Immunol.

[65] Hongbo C. Regulation and function of mTOR signalling in T cell fate decisions. Nat Rev Immunol. 2012;5(12):325–38. 10.1038/nri3198 PMC3417069 22517423

[66] Cluxton D, Petrasca A, Moran B, Fletcher JM (2019). Differential regulation of human Treg and Th17 cells by fatty acid synthesis and glycolysis. Front Immunol.

[67] Polzonetti V, Pucciarelli S, Vincenzetti S, Polidori P. Dietary Intake of Vitamin D from Dairy Products Reduces the Risk of Osteoporosis. Nutrients. 2020;12(6):1743. 10.3390/nu12061743 PMC7353177 32532150

[68] Dar HY, Singh A, Shukla P, Anupam R, Mondal RK, Mishra PK (2018). High dietary salt intake correlates with modulated Th17-Treg cell balance resulting in enhanced bone loss and impaired bone-microarchitecture in male mice. Sci Rep.

[69] Yang YH, Istomine R, Alvarez F, Al-Aubodah TA, Shi XQ, Takano T (2020). Salt sensing by serum/glucocorticoid-regulated kinase 1 promotes Th17-like inflammatory adaptation of Foxp3(+) regulatory T cells. Cell Rep.

[70] Wu L, Luthringer BJC, Feyerabend F, Zhang Z, Machens HG, Maeda M (2017). Increased levels of sodium chloride directly increase osteoclastic differentiation and resorption in mice and men. Osteoporos Int.

[71] Schröder A, Neubert P, Titze J, Bozec A, Neuhofer W, Proff P, et al. Osteoprotective action of low-salt diet requires myeloid cell–derived NFAT5. JCI Insight. 2019;4(23). 10.1172/jci.insight.127868 PMC6962031 31801906

[72] Antonios TF, Macgregor GA (1997). Salt--more adverse effects. Am J Hypertens.

[73] Pao-Hwa L, Fiona G, Appel LJ, Mikel A, Arline B, Patrick G (2003). The DASH diet and sodium reduction improve markers of bone turnover and calcium metabolism in adults. J Nutr.

[74] Hu ED, Chen DZ, Wu JL, Lu FB, Chen L, Zheng MH (2018). High fiber dietary and sodium butyrate attenuate experimental autoimmune hepatitis through regulation of immune regulatory cells and intestinal barrier. Cell Immunol.

[75] Furusawa Y, Obata Y, Fukuda S, Endo TA, Nakato G, Takahashi D (2013). Commensal microbe-derived butyrate induces the differentiation of colonic regulatory T cells. Nature..

[76] Singh N, Gurav A, Sivaprakasam S, Brady E, Padia R, Shi H (2014). Activation of Gpr109a, receptor for niacin and the commensal metabolite butyrate, suppresses colonic inflammation and carcinogenesis. Immunity..

[77] Smith PM, Howitt MR, Panikov N, Michaud M, Gallini CA, Bohlooly YM (2013). The microbial metabolites, short-chain fatty acids, regulate colonic Treg cell homeostasis. Science..

[78] Haase S, Haghikia A, Wilck N, Muller DN, Linker RA (2018). Impacts of microbiome metabolites on immune regulation and autoimmunity. Immunology..

[79] Mucida D, Park Y, Kim G, Turovskaya O, Scott I, Kronenberg M (2007). Reciprocal TH17 and regulatory T cell differentiation mediated by retinoic acid. Science..

[80] Sun CM, Hall JA, Blank RB, Bouladoux N, Oukka M, Mora JR (2007). Small intestine lamina propria dendritic cells promote de novo generation of Foxp3 T reg cells via retinoic acid. J Exp Med.

[81] Schilderink R, Verseijden C, Seppen J, Muncan V, van den Brink GR, Lambers TT (2016). The SCFA butyrate stimulates the epithelial production of retinoic acid via inhibition of epithelial HDAC. Am J Physiol Gastrointest Liver Physiol.

[82] Tanoue T, Atarashi K, Honda K (2016). Development and maintenance of intestinal regulatory T cells. Nat Rev Immunol..

[83] Caspar O. MUCOSAL IMMUNOLOGY. The microbiota regulates type 2 immunity through RORγt? T cells. Science (New York, NY). 2015;6251(349):989–93. 10.1126/science.aac4263 26160380

[84] Peter J, Sabu V, Aswathy IS, Krishnan S, Lal Preethi SS, Simon M, et al. Dietary amaranths modulate the immune response via balancing Th1/Th2 and Th17/Treg response in collagen-induced arthritis. Mol Cell Biochem. 2020. 10.1007/s11010-020-03783-x 32529499

[85] Ibáñez L, Rouleau M, Wakkach A, Blin-Wakkach C (2019). Gut microbiome and bone. Joint Bone Spine.

[86] Estrada JA, Contreras I. Nutritional Modulation of Immune and Central Nervous System Homeostasis: The Role of Diet in Development of Neuroinflammation and Neurological Disease. Nutrients. 2019;11(5):1076. 10.3390/nu11051076 PMC6566411 31096592

[87] Donkor ON, Ravikumar M, Proudfoot O, Day SL, Apostolopoulos V, Paukovics G (2012). Cytokine profile and induction of T helper type 17 and regulatory T cells by human peripheral mononuclear cells after microbial exposure. Clin Exp Immunol.

[88] Yu R, Zuo F, Ma H, Chen S. Exopolysaccharide-Producing Bifidobacterium adolescentis Strains with Similar Adhesion Property Induce Differential Regulation of Inflammatory Immune Response in Treg/Th17 Axis of DSS-Colitis Mice. Nutrients. 2019;11(4):782. 10.3390/nu11040782 PMC6520857 30987344

[89] Onuora S (2016). Autoimmunity: human gut bacteria induce TH17 cells. Nat Rev Rheumatol.

[90] Cheng J, Guan, Chen Q, Shujiao M (2019). The Th17/Treg Cell Balance: A Gut Microbiota-Modulated Story. Microorganisms.

[91] Dar HY, Pal S, Shukla P, Mishra PK, Tomar GB, Chattopadhyay N (2018). Bacillus clausii inhibits bone loss by skewing Treg-Th17 cell equilibrium in postmenopausal osteoporotic mice model. Nutrition..

[92] Jang YJ, Kim WK, Han DH, Lee K, Ko G (2019). Lactobacillus fermentum species ameliorate dextran sulfate sodium-induced colitis by regulating the immune response and altering gut microbiota. Gut Microbes.

[93] Quintana FJ, Basso AS, Iglesias AH, Korn T, Farez MF, Bettelli E (2008). Control of T (reg) and T(H)17 cell differentiation by the aryl hydrocarbon receptor. Nature..

[94] Xiong L, Dean J, Fu Z, Oliff K, Bostick J, Ye J (2020). Ahr-Foxp3-RORγt axis controls gut homing of CD4 + T cells by regulating GPR15. Sci Immunol.

[95] Carriche GM, Almeida L, Stuve P, Velasquez L, Dhillon-LaBrooy A, Roy U, et al. Regulating T-cell differentiation through the polyamine spermidine. J Allergy Clin Immunol. 2020. 10.1016/j.jaci.2020.04.037 32407834

[96] Kochetkova I, Thornburg T, Callis G, Holderness K, Maddaloni M, Pascual DW (2014). Oral Escherichia coli colonization factor antigen I fimbriae ameliorate arthritis via IL-35, not IL-27. J Immunol.

[97] Ding K, Hua F, Ding W (2020). Gut microbiome and osteoporosis. Aging Dis.

